# Hypofractionated radiotherapy has the potential for second cancer reduction

**DOI:** 10.1186/1742-4682-7-4

**Published:** 2010-02-11

**Authors:** Uwe Schneider, Jürgen Besserer, Andreas Mack

**Affiliations:** 1 Radiotherapy Hirslanden AG, Institute for Radiotherapy, Rain 34, 5001 Aarau, Switzerland; 2 Vetsuisse Facutly, University of Zürich, Winterthurerstrasse 260, 8057 Zürich, Switzerland

## Abstract

**Background and Purpose:**

A model for carcinoma and sarcoma induction was used to study the dependence of carcinogenesis after radiotherapy on fractionation.

**Materials and methods:**

A cancer induction model for radiotherapy doses including fractionation was used to model carcinoma and sarcoma induction after a radiation treatment. For different fractionation schemes the dose response relationships were obtained. Tumor induction was studied as a function of dose per fraction.

**Results:**

If it is assumed that the tumor is treated up to the same biologically equivalent dose it was found that large dose fractions could decrease second cancer induction. The risk decreases approximately linear with increasing fraction size and is more pronounced for sarcoma induction. Carcinoma induction decreases by around 10% per 1 Gy increase in fraction dose. Sarcoma risk is decreased by about 15% per 1 Gy increase in fractionation. It is also found that tissue which is irradiated using large dose fractions to dose levels lower than 10% of the target dose potentially develop less sarcomas when compared to tissues irradiated to all dose levels. This is not observed for carcinoma induction.

**Conclusions:**

It was found that carcinoma as well as sarcoma risk decreases with increasing fractionation dose. The reduction of sarcoma risk is even more pronounced than carcinoma risk. Hypofractionation is potentially beneficial with regard to second cancer induction.

## Introduction

Although the increased risk for radiotherapy patients to develop a secondary malignancy is small, it is statistically significant, in particular for long time survivors of treatment. As a consequence of better radiation treatment modalities available, cancer cure rates have increased. As a result, there are now many long term survivors of cancer who are at risk of late effects of therapy, including secondary cancers.

Hypo-fractionated treatment schedules are proposed for several types of cancers, including cancer of the breast [1-4], prostate [5-7] and lung [8,9]. Although such treatment options are still related to major concerns such as the uncertainty to predict the correct complication probabilities of normal tissues and control probabilities for tumor tissue it might be of interest to study their impact on radiation induced cancer.

Estimates of radiation carcinogenesis after radiotherapy can be based on epidemiological studies of patients treated with old techniques. However, most of the epidemiological studies, which are published in a large number, don't provide a correlation of cancer induction with dose. Unfortunately, if a dose correlation is deduced, cancer induction is usually related to integral dose or average organ dose and thus implies a linear dose-response relationship. Thus, such data cannot be used directly to obtain non-linear dose-response relationships for radiotherapy. Therefore, as an alternative cancer risk models can be used to estimate second malignancies after radiotherapy. Those models can be validated with epidemiologic studies.

Sachs and Brenner [10] developed a discrete algebraic model of dose-dependent cancer risk, incorporating cell killing and proliferation/repopulation effects. In this report we use a model based on a continuous approach with a dose variation from zero to the total delivered dose which leads to an analytic representation of cancer risk. The model includes fractionation effects and distinguishes between carcinoma and sarcoma induction.

## Materials and methods

**Figure 1:**
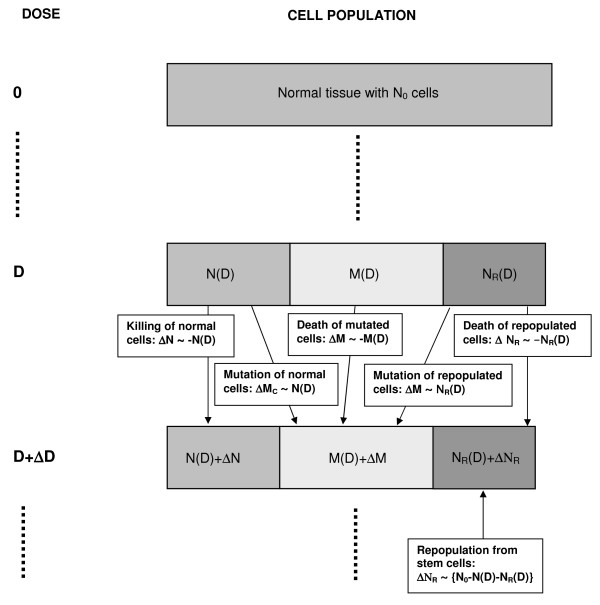
**Diagram of the cell kinetics which were assumed in the presented model**. *M *indicates the number of mutated cells for both sarcoma and carcinoma induction and *M*_*C *_indicates mutated cells only for the case of carcinoma induction.

For modeling cancer risk after fractionated radiotherapy it is assumed that the tissue or organ of interest consists of *N*_0 _cells before it is irradiated. At this stage there is no distinction between cells which represent a particular function and do not divide and stem cells which are dividing. The tissue is now irradiated with a fractionated treatment schedule of equal dose fractions *d *up to a dose *D*. The number of original cells after irradiation is reduced by cell kill. A number of *N *cells survive one dose fraction. Cell kill is proportional to *α' *which is defined using the linear quadratic model(1)

where *D*_*T *_and *d*_*T *_is the prescribed dose to the target volume with the corresponding fractionation dose, respectively. Fig. 1 shows a compartmental diagram of the modeled processes. It is further assumed for this work, that the number of killed original tissue cells *N*_0_-*N *is replaced by a number of new cells *N*_*R *_with a repopulation rate which is proportional to *N*_0_-*N-N*_*R*_. Here it is assumed that the repopulation kinetics of repopulated cells will follow the same basic patterns as those of normal cells. Cells which were irradiated can be mutated and have the potential to develop a tumor. In the context of this work the word "mutation" is used as a synonym for each cell transformation which results in a new tumor cell. In fact the development of a tumor usually implies several mutations. The mutational process is modeled here according to the linear-no-threshold model and thus cancer risk originating from an irradiation with one dose fraction *d *is taken proportional to *μ *which is the slope of cancer induction from the linear-no-threshold model which is mainly based on the data of the A-bomb survivors. If is now assumed that the number of involved cells is treated as a continuous function of dose a system of differential equations derived from the cell kinetics as described in Fig. 1 can be solved [11]. The excess absolute risk for carcinoma induction is then(2)

and for sarcoma induction(3)

where *R *is the repopulation parameter which characterizes the ability of the tissue to repopulate. Risk was computed for different fractionation schemes using Equs.2 and 3.

## Results

**Figure 2:**
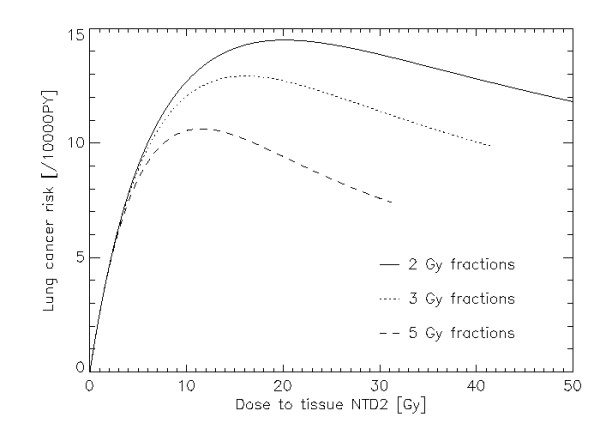
**Plot of lung cancer risk per 10000 persons per year as a function of absorbed dose in normal tissue**. The solid, dotted and dashed lines represent 2 Gy, 3 Gy and 5 Gy fractions. The total dose to the target was 50 Gy for the 2 Gy fractionation schedule and was adjusted for the other fraction sizes (by applying the LQ-model) to achieve the same biological effect. The model parameters were for the initial slope *μ *= 2.8 per 10000 PY per Gy per year, *α *= 0.09 Gy^-1^, *α/β *= 3 Gy and *R *= 0.7.

Fig. 2 shows lung carcinoma risk as a function of equivalent dose corresponding to 2 Gy fractions (NTD2). NTD2 is used here since different fractionation schedules result in different biological effectiveness for the same physical dose. Thus in radiotherapy it is convenient to use the dose which would result in the same biological effect as a 2 Gy fractionation schedule (NTD2). The solid line represents carcinoma risk for a treatment up to 50 Gy target dose in 2 Gy fractions, the dotted line up to 42 Gy in 3 Gy fractions and the dashed line up to 30 Gy in 5 Gy fractions. Computations were done using an *α/β *= 3 Gy. It should be noted here that the dose plotted in Fig. 2 is absorbed dose in healthy tissue. Therefore a data point on the dose axis corresponds to different daily dose fraction. For example the 25 Gy data point would correspond to 1.0 Gy, 1.8 Gy and 4.2 Gy daily dose fractions for the 2 Gy, 3 Gy and 5 Gy target fractionation schedule. The initial slope *μ *for this figure was obtained from the analysis of the A-bomb survivors [12,13]. The A-bomb data were used for an age at exposure of 48.5 years and attained age of 59 years, since those data are characteristic for a population of treated Hodgkin's patients [14]. This example is chosen as Hodgkin's patients which were treated with radiation are at a significant enhanced risk for lung cancer [14]. The initial slope *μ *is then 2.8 per 10000 PY per Gy per year. The other parameters were *α *= 0.10 Gy^-1^, *α/β *= 3 Gy and *R *= 0.7. Fig. 2 shows a significant advantage for large dose fractionations with respect to cancer induction.

**Figure 3:**
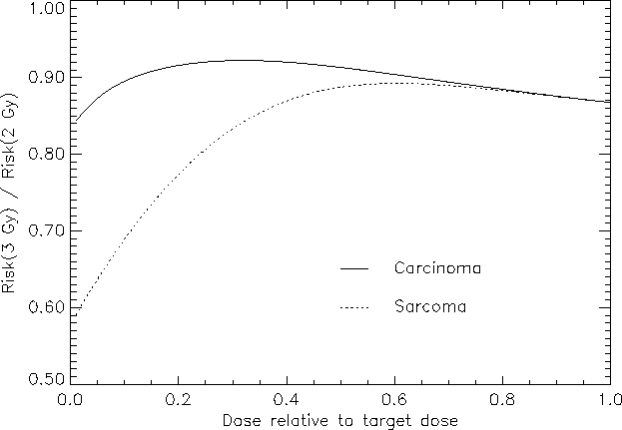
**Carcinoma (solid line) and sarcoma risk (dotted line) for a 3 Gy fractionation schedule relative to a 2 Gy fractionation**. The model parameters are *α *= 0.09 Gy^-1^, *α/β *= 3 Gy and *R *= 0.7

The risk ratio for a 3 Gy fractionation relative to a 2 Gy schedule is shown in Fig. 3 both for carcinoma and sarcoma induction as a function of dose relative to the target dose. Carcinoma risk reduction is for the whole dose range more or less constant in contrast to sarcoma risk reduction which varies significantly with dose and is larger for tissue which receives only small dose. It should be noted here that the relative risks (with regard to fractionation) are independent of the initial slope *μ *of the dose response curve since *μ *cancels out when using ratios of Equs. 2 and 3, respectively. Thus the relative risks between different fractionation schedules are in a first approximation independent of the type of cancer.

**Figure 4:**
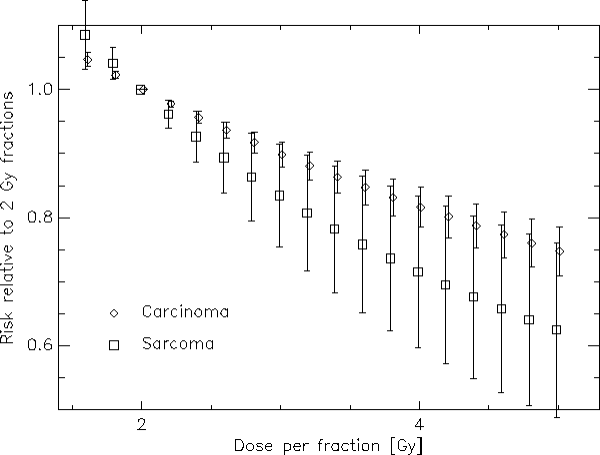
**Risk ratio between treatments with different fractionations relative to a 2 Gy fractionation schedule plotted as a function of dose per fraction**. The diamonds represent carcinoma and the squares sarcoma induction. The bars represent the variation of risk difference for the different dose levels including tissues which receive nearly no dose up to the target dose.

Fig. 4 shows the risk relative to a 2 Gy fractionation scheme quantitatively as a function of fraction size for a 50 Gy NTD2 treatment. The error bars represent the variation of the risk ratio over the complete dose range. A 3 Gy fractionation schedule for example could result in a 10% reduction of second cancers relative to a 2 Gy fractionated treatment. It is also shown that this advantage is more distinct for sarcoma induction than for carcinoma induction. Since the risk advantage is approximately a linear function of fractionation dose a simple rule yields a 10% reduction in second carcinoma risk per 1 Gy increase in fractionation dose and a 13%/Gy reduction for sarcoma (*α *= 0.10 Gy^-1^, *α/β *= 3 Gy, *R *= 0.7). This risk reduction is dependent on the chosen model parameters. A variation of the repopulation parameter *R *between 0 and 1 yields a variation of carcinoma risk reduction between 5.2% and 10.3% and of sarcoma risk reduction between 12.5% and 13.8%. A variation of *α *between 0.05 and 0.15 yields a variation of carcinoma risk reduction between 8.3% and 12.4% and of sarcoma risk reduction between 12.0% and 14.5%. As a conclusion the fractionation advantage is slightly dependent on the model parameters.

**Figure 5:**
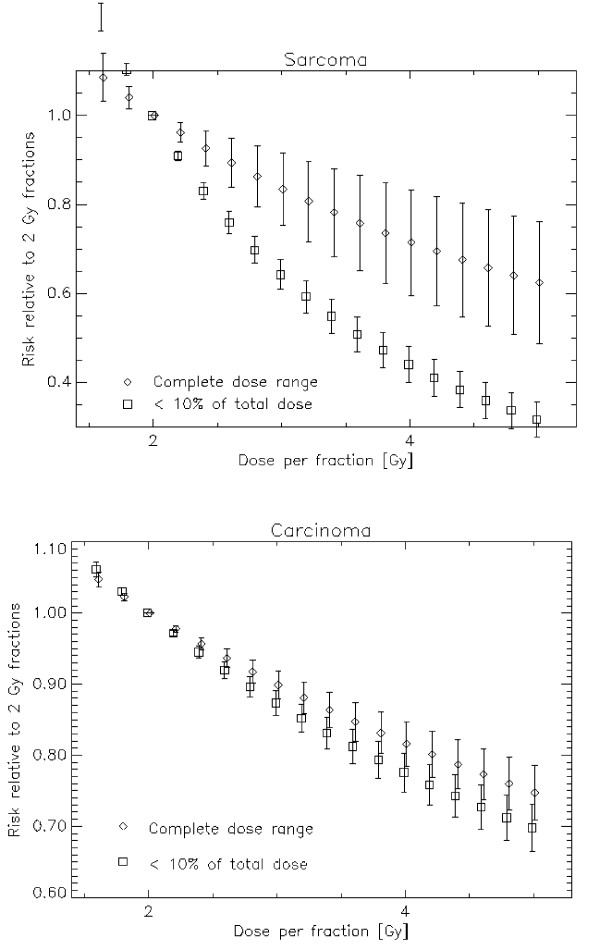
**Risk ratio between a treatments with different fractionations relative to a 2 Gy fractionation schedule and plotted as a function of dose per fraction**. For sarcoma in a) and for carcinoma in b). The diamonds represent the average over the whole dose range and the squares are averages up to 10% of the target dose which is 5 Gy NTD2 in this example. The bars represent the variation of risk ratio over the dose range.

Figs. 5a and 5b show second cancer risk relative to a 2 Gy fractionation as a function of fractionation dose for sarcoma and carcinoma, respectively. In this plot the diamonds represent the average dose advantage for all dose levels (as in Fig. 4). The squares represent the dose advantage only for such tissues which receive less than 10% of the prescribed dose, which is in this example 5 Gy (NTD2). Clearly, it can be observed that the advantage of larger fraction sizes with respect to sarcoma induction might be more significant for low dose volumes. On the other hand the risk reduction for carcinomas is approximately independent of the irradiated dose.

## Discussion

In this report a cancer induction model for the radiotherapy dose range was used. Several assumptions had to be made to simplify the biological processes leading to cancer induction [11]. This includes the design of tissues, the repopulation process and processes which result in the formation of a tumor cell. This was done to keep the number of model parameters at a minimum. However, this is associated with uncertainties. It was for example suggested [15] that some radiotherapy-induced second cancers can be the consequence of late normal tissue damage leading to a precancerous lesion. Such a mechanism is not included in the present model.

Another assumption of the presented model is that single dose fractions of a radiotherapy treatment are treated independently. Therefore the linear-no-threshold theory for cancer induction could be applied to each single dose fraction. Although this may be valid for a single exposure lower than 3 Gy, for fractionation schedules with single doses of more than 3 Gy the model must be used with care. In addition it is not clear whether it can be applied for dose fractions which are separated by days when in fact not all cells are fully repaired.

Many problems and uncertainties are involved in modeling the underlying biology of radiation induced cancer. However, since very little is currently known about the shape of dose-response relationships for radiation-induced cancer in the radiotherapy dose range, this approach can be used to look at least qualitatively at the fractionation dependence of cancer induction for carcinomas and sarcomas separately.

As the results of this report are expressed in terms of EAR it is also difficult to compare them with the findings of Sachs and Brenner [10] who fitted an algebraic model of cancer induction to lung cancer risk.

If the fraction size is increased while keeping the total dose at a level which corresponds to the same biological response (LQ-model) it is shown that cancer induction is decreasing linearly. This decrease occurs for both carcinoma and sarcoma induction, however, the effect is more pronounced for sarcoma induction. Quantitatively a reduction of around 5% to 15% is expected while increasing the fraction size from 2 Gy to 3 Gy. As a consequence hypo-fractionated treatment techniques are with regard to cancer induction advantageous when compared to conventional fractionation schedules, as for example in prostate radiotherapy where hypofractionation is realized with intensity modulated treatment techniques (IMRT). However, if both a hypofractionated treatment schedule and a new treatment technique is applied (for example a cyberknife treatment), risk must be analysed in detail. The dose distributions resulting from cyberknife treatments are significantly different from conventional therapy and hence the changes in risk with regard to dose distribution might balance the implications of changes in the fractionation.

## Conclusions

A cancer induction model for fractionated radiotherapy was used to investigate the impact of different fractionation schedules on second cancer risk.

It was found that carcinoma as well as sarcoma risk decreases with increasing fractionation dose. This decrease is nearly linear with fractionation dose and is more distinct for sarcoma induction. It was also shown that the risk advantage for the sarcoma induction is significantly dependent on the dose in the tissue and is more enhanced for tissue irradiated with low dose (< 10% of the prescribed dose).

## Competing interests

The authors state that there is no conflict of interest for the authors or the author's institution and that they have no financial or personal relationships that inappropriately influence their actions. They have no dual commitments, competing interests, competing loyalties, employment, consultancies, stock ownership, honoraria, or paid expert testimony.

## Authors' contributions

US contributed in constructing the model. JB analysed the biological modeling. AM was involved in applying the results to Cyberknife treatments. All authors read and approved the final manuscript.
